# Diagnosis of a giant gastric subepithelial lesion using a dual-frequency ultrasonic miniprobe

**DOI:** 10.1055/a-2192-0277

**Published:** 2023-11-20

**Authors:** Suhuan Liao, Weiguang Qiao, Silin Huang, Guang Yang, Ronggang Zhang, Weiwen Wu, Xiaosong Bai

**Affiliations:** 1 Department of Gastroenterology, South China Hospital, Medical School, Shenzhen University, Shenzhen, China; 2 Guangdong Provincial Key Laboratory of Gastroenterology, Department of Gastroenterology, Nanfang Hospital, Southern Medical University, Guangzhou, China; 3 InnerMedical Co., Ltd., Shenzhen, China

A 43-year-old woman was referred for endoscopic resection after detection of a giant subepithelial lesion (SEL) during routine physical assessment. The lesion was approximately 50 × 25 mm in size and was located in the posterior wall of the gastric fundus ( Fig. 1 ). A comprehensive assessment of the origin and characteristics of the lesion was performed via a dual-frequency ultrasonic miniprobe (InnerMedical Co., Ltd., Shenzhen, China) ( Fig. 2 , Video 1 ).

**Fig. 1:**
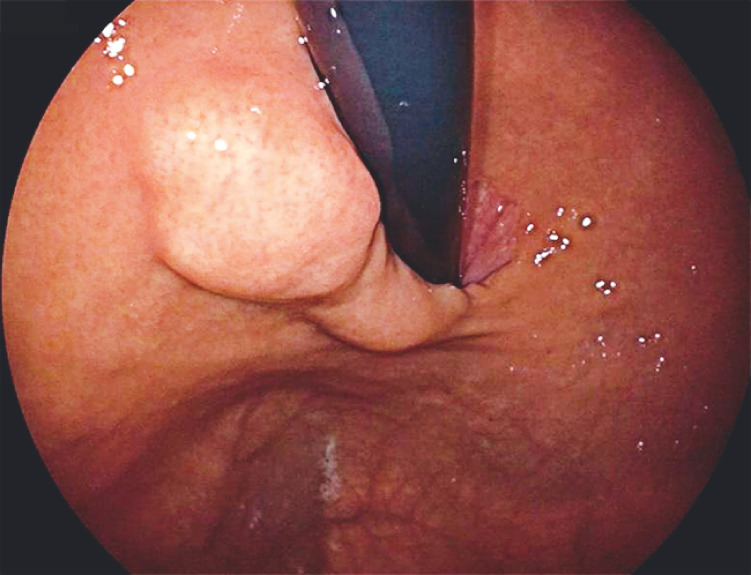
A subepithelial lesion, size approximately 50 × 25 mm, was detected in the gastric fundus. The surface of the lesion showed irregular contours, indeterminate borders, and restricted mobility.

**Fig. 2:**
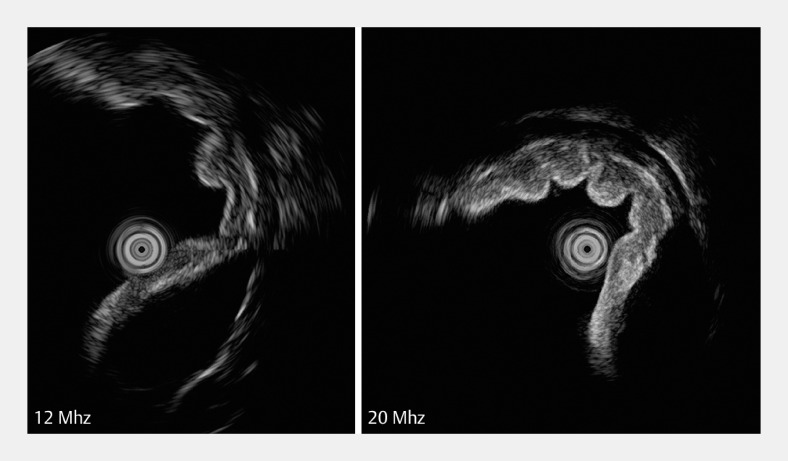
The dual-frequency miniprobe integrates both 12 MHz and 20 MHz into one device.

Application of a new-generation dual-frequency ultrasonic miniprobe in diagnosing giant gastric subepithelial lesions.Video 1

Initially, the 20 MHz setting of the probe elucidated the lesion origin at the muscularis propria, revealing hypoechoic alterations ( Fig. 3 a). In pursuit of additional data pertaining to the lesion and its adjacent structures, the probe frequency was effortlessly adjusted to 12 MHz by pressing a button. The 12 MHz setting markedly enhanced the lesion’s echogenic profile in the distal region. The lesion was characterized by its inward growth, marked by hypoechoic features interspersed with internal echogenic debris ( Fig. 3 b). The dual-frequency ultrasonic miniprobe findings were indicative of a leiomyoma, which prompted subsequent endoscopic submucosal excavation (ESE) ( Fig. 4 a–c) for complete resection. Postoperative pathological analysis confirmed the diagnosis of leiomyoma ( Fig. 4 d).

**Fig. 3:**
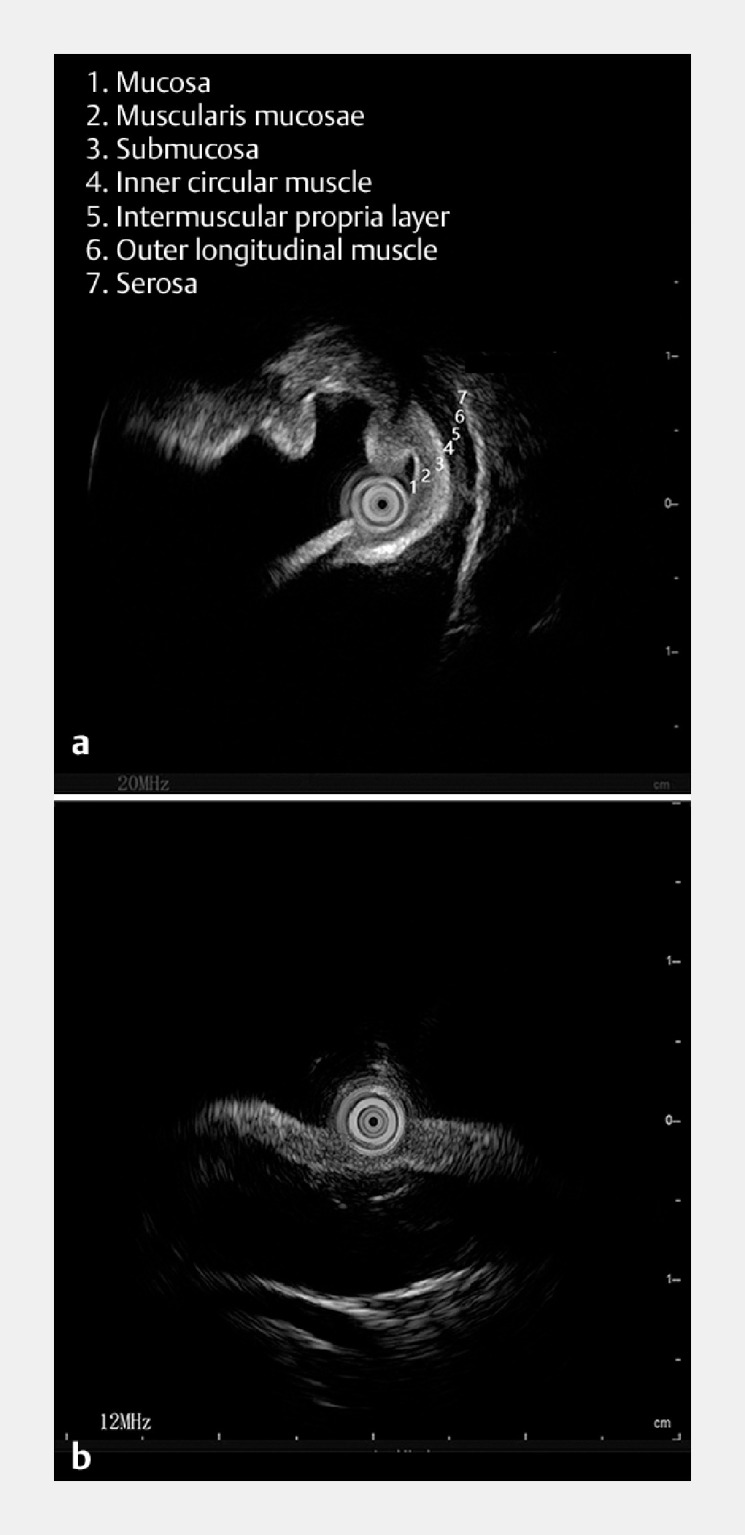
Utilization of the dual-frequency ultrasonic miniprobe in the diagnosis of giant gastric subepithelial lesions. **a** The 20 MHz setting of the probe elucidated the lesion origin at the muscularis propria layer, and displayed hypoechoic alterations. **b** Under the 12 MHz setting, the lesion manifested inward proliferation, distinguished by hypoechoic shifts with incorporated echogenic detritus.

**Fig. 4:**
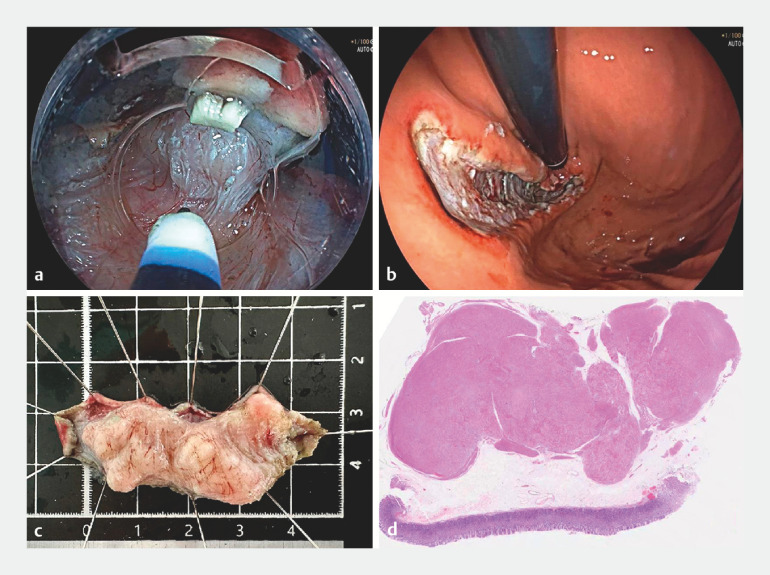
Endoscopic submucosal excavation (ESE) of the tumor. **a** The subepithelial lesion, approximating 50 × 25 mm, was identified within the gastric fundus. The surface of the lesion showed irregular contours, indeterminate borders, and restricted mobility. **b** Surgical wound post-excision. **c** Preservation of the excised specimen. **d** Histopathological analysis indicating the presence of a leiomyoma.

For SELs, radial endoscopic ultrasound or miniprobe have become widely adopted across the globe to ascertain the depth of the lesion and to predict its nature 1 2 3 . Compared with radial endoscopic ultrasound, miniprobes are more cost-effective and offer greater ease in terms of learning and operation. Additionally, miniprobes utilize higher frequencies, enabling superior visualization of the origin and extent of SELs 3 4 . Unlike traditional miniprobes, this innovative miniprobe integrates both 12 MHz and 20 MHz into a single device. Users can seamlessly toggle between frequencies via a button, eliminating the need to change endoscopes or probes, and consequently saving valuable diagnostic time. Despite the absence of Doppler functionality in the current dual-frequency miniprobe, which precludes detailed vascular assessment, it is not deemed necessary in this instance as the lesion is solid. To the best of our knowledge this case represents the first use of a dual-frequency ultrasonic miniprobe to diagnose a giant gastric SEL, establishing an invaluable foundation for future clinical applications.

Endoscopy_UCTN_Code_TTT_1AS_2AB

## Notes

Endoscopy E-Videos https://eref.thieme.de/e-videos
*E-Videos* is an open access online section of the journal *Endoscopy* , reporting on interesting cases and new techniques in gastroenterological endoscopy. All papers include a high-quality video and are published with a Creative Commons CC-BY license. Endoscopy E-Videos qualify for HINARI discounts and waivers and eligibility is automatically checked during the submission process. We grant 100% waivers to articles whose corresponding authors are based in Group A countries and 50% waivers to those who are based in Group B countries as classified by Research4Life (see: https://www.research4life.org/access/eligibility/ ). This section has its own submission website at https://mc.manuscriptcentral.com/e-videos .
